# Supplementary material for: Extracts of Digested Berries Increase the Survival of Saccharomyces cerevisiae during H2O2 Induced Oxidative Stress
Source: Molecules. 2021 Feb 18;26(4):1057. doi: 10.3390/molecules26041057 (PMC7922075; doi:10.3390/molecules26041057)
Supplement: Supplementary file 1 [file molecules-26-01057-s001.pdf]

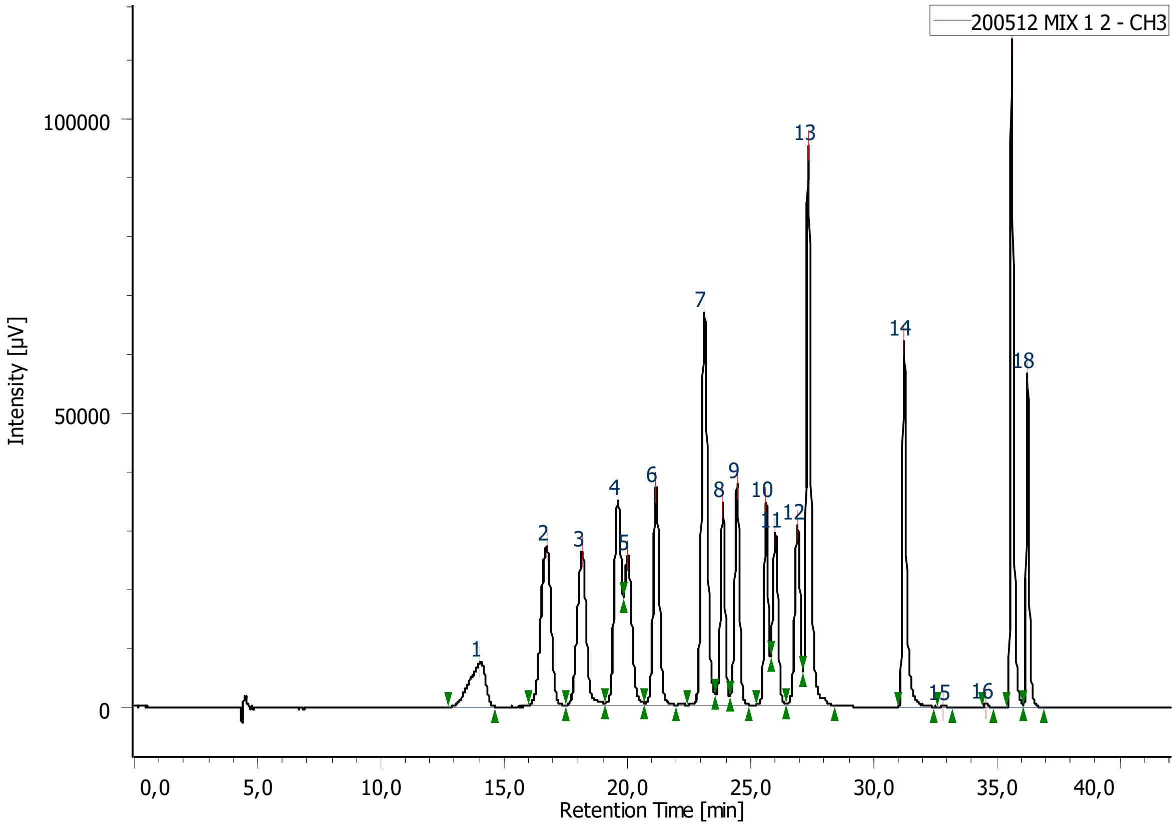

Fig. S1A. Example of a chromatogram for the anthocyanin standards used in this study. Peaks correspond to the following anthocyanins: (1) cyanidin-3,5-diglucoside, (2) pelargonidin-3-O-rutinoside, (3) delphinidin-3-O-galactoside, (4) delphinidin-3-glucoside, (5) cyanidin-3-O-galactoside, (6) malvidin-3,5-diglucoside; (7) delphinidin-3-O-rutinoside, (8) Cyanidin-3-glucoside, (9) cyanidin-3-O-rutinosoide, (10) cyanidin-3-O-arabinose, (11) pelargonidin-3-glucoside, (12) peonidin-3-O-galactoside, (13) pelargonidin, (14) malvidin-3-O-galactoside, (15) malvidin-3-glucoside, (16) peonidin-3-O-arabinoside, (17) delphinidin, (18) cyanidine.

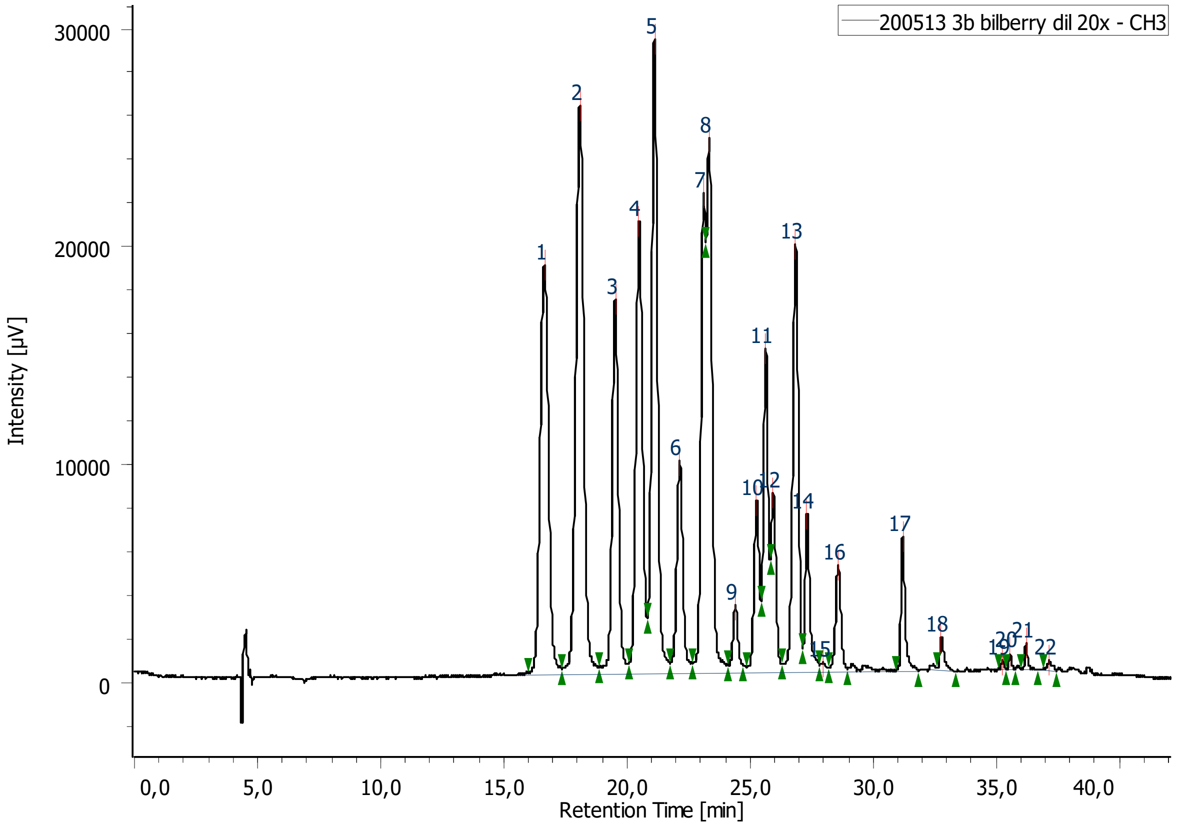

Fig.S1B. Example of a chromatogram for bilberry extract <u>before in vitro digestion.</u> Peaks correspond to the following anthocyanins: (1) cyanidin-3,5-diglucoside, (2) pelargonidin-3-O-rutinoside, (3) delphinidin-3-O-galactoside, (4) delphinidin-3-glucoside, (5) cyanidin-3-O-galactoside, (6) malvidin-3,5-diglucoside; (7) delphinidin-3-O-rutinoside, (8) Cyanidin-3-glucoside, (9) cyanidin-3-O-rutinosoide, (10) cyanidin-3-O-arabinose, (11) pelargonidin-3-glucoside, (12) peonidin-3-O-galactoside, (13) pelargonidin, (14) malvidin-3-O-galactoside, (15) malvidin-3-glucoside, (16) peonidin-3-O-arabinoside, (17) delphinidin, (18) cyanidine.

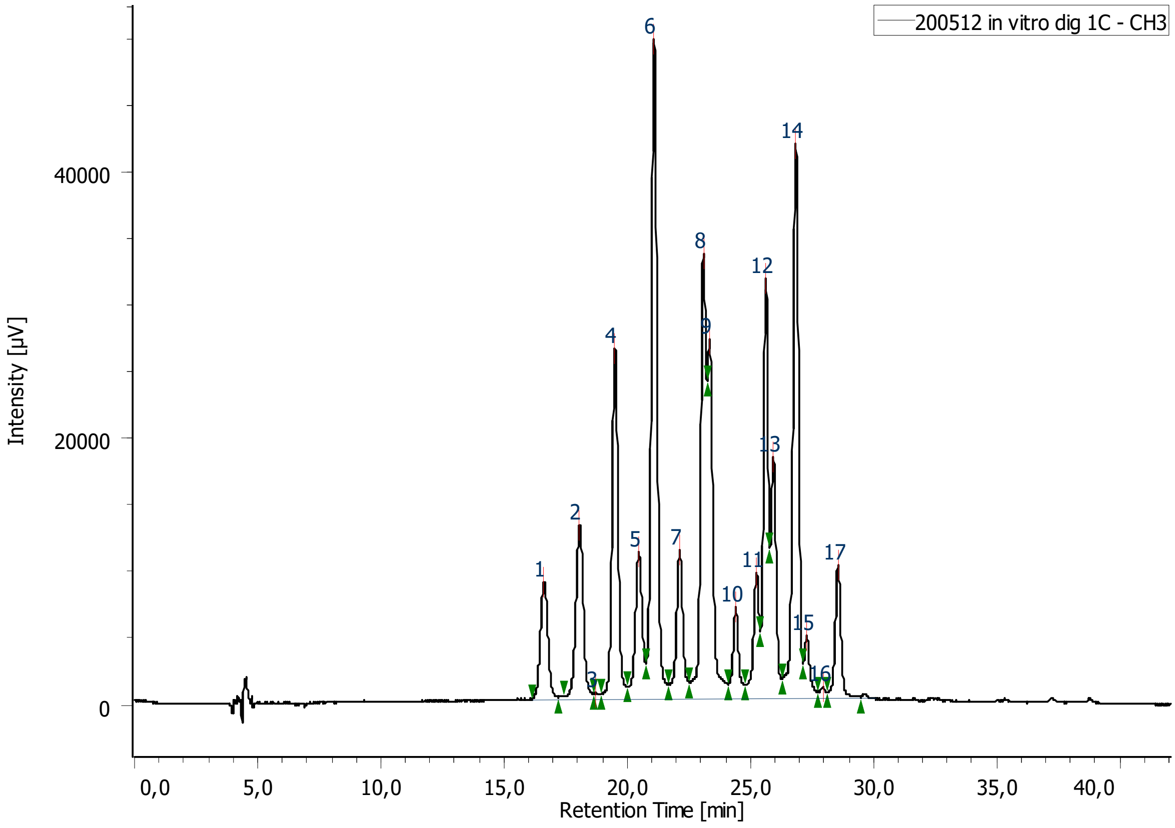

Fig. S1C. Example of a chromatogram for bilberry extract after in vitro digestion. Peaks correspond to the following anthocyanins: (1) cyanidin-3,5-diglucoside, (2) pelargonidin-3-O-rutinoside, (3) delphinidin-3-O-galactoside, (4) delphinidin-3-glucoside, (5) cyanidin-3-O-galactoside, (6) malvidin-3,5-diglucoside; (7) delphinidin-3-O-rutinoside, (8) Cyanidin-3-glucoside, (9) cyanidin-3-O-rutinosoide, (10) cyanidin-3-O-arabinose, (11) pelargonidin-3-glucoside, (12) peonidin-3-O-galactoside, (13) pelargonidin, (14) malvidin-3-O-galactoside, (15) malvidin-3-glucoside, (16) peonidin-3-O-arabinoside, (17) delphinidin.

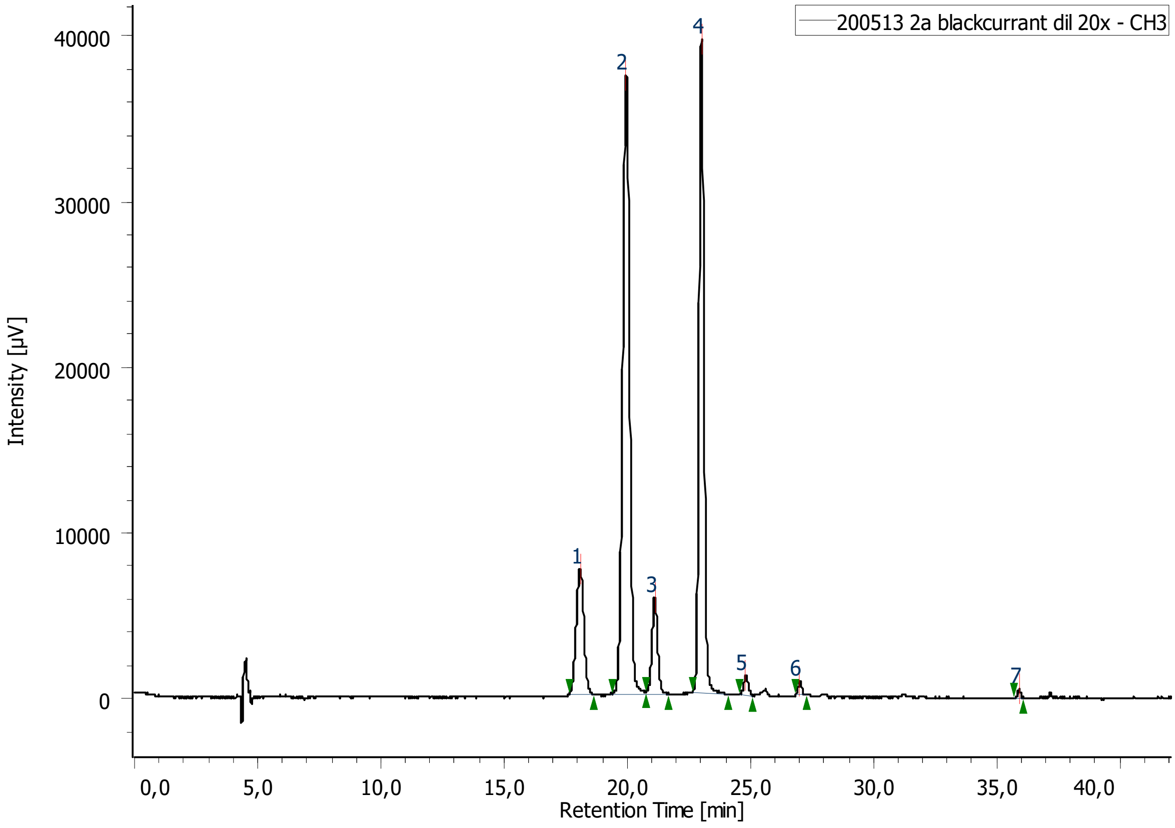

Fig. S1D. Example of a chromatogram for blackcurrant extract before in vitro digestion. Peaks correspond to the following anthocyanins: (1) cyanidin-3,5-diglucoside, (2) pelargonidin-3-O-rutinoside, (3) delphinidin-3-O-galactoside, (4) delphinidin-3-glucoside, (5) cyanidin-3-O-galactoside, (6) malvidin-3,5-diglucoside; (7) delphinidin-3-O-rutinoside.

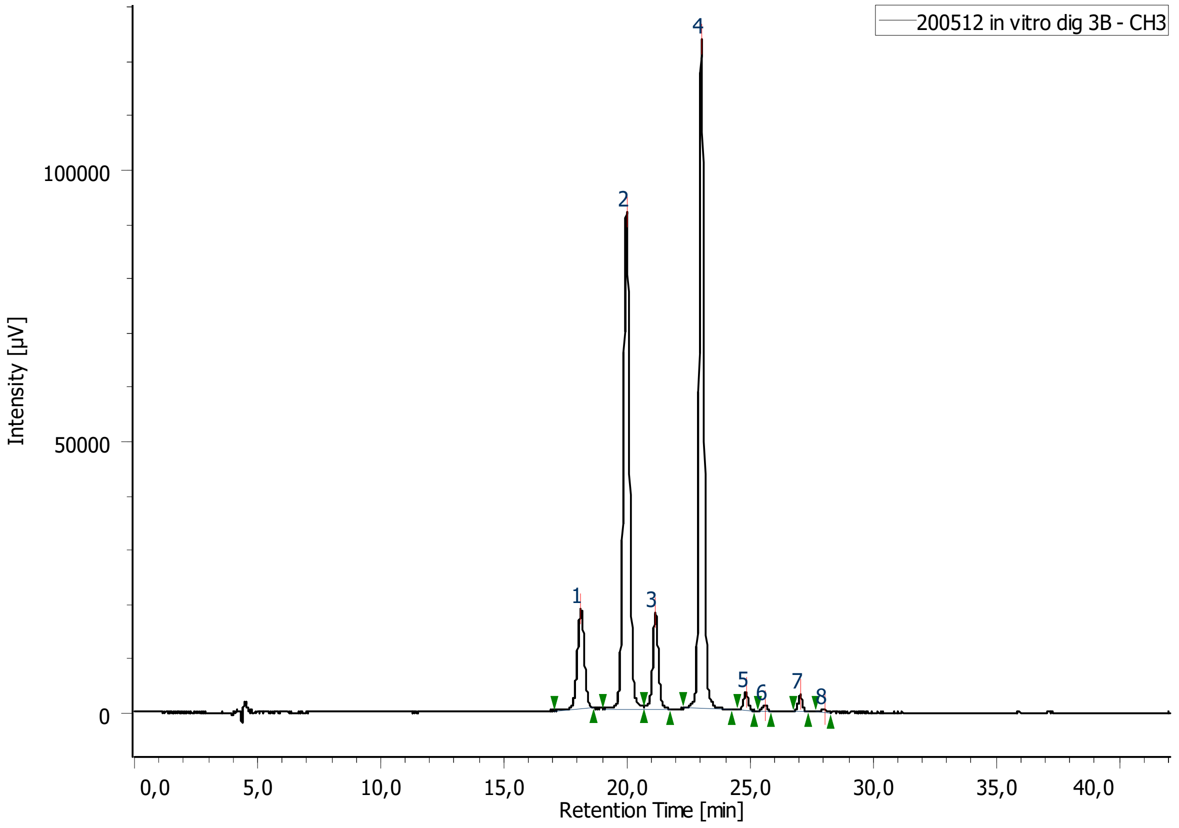

Fig. S1E. Example of a chromatogram for blackcurrant extract after in vitro digestion. Peaks correspond to the following anthocyanins: (1) cyanidin-3,5-diglucoside, (2) pelargonidin-3-O-rutinoside, (3) delphinidin-3-O-galactoside, (4) delphinidin-3-glucoside, (5) cyanidin-3-O-galactoside, (6) malvidin-3,5-diglucoside; (7) delphinidin-3-O-rutinoside, (8) Cyanidin-3-glucoside.

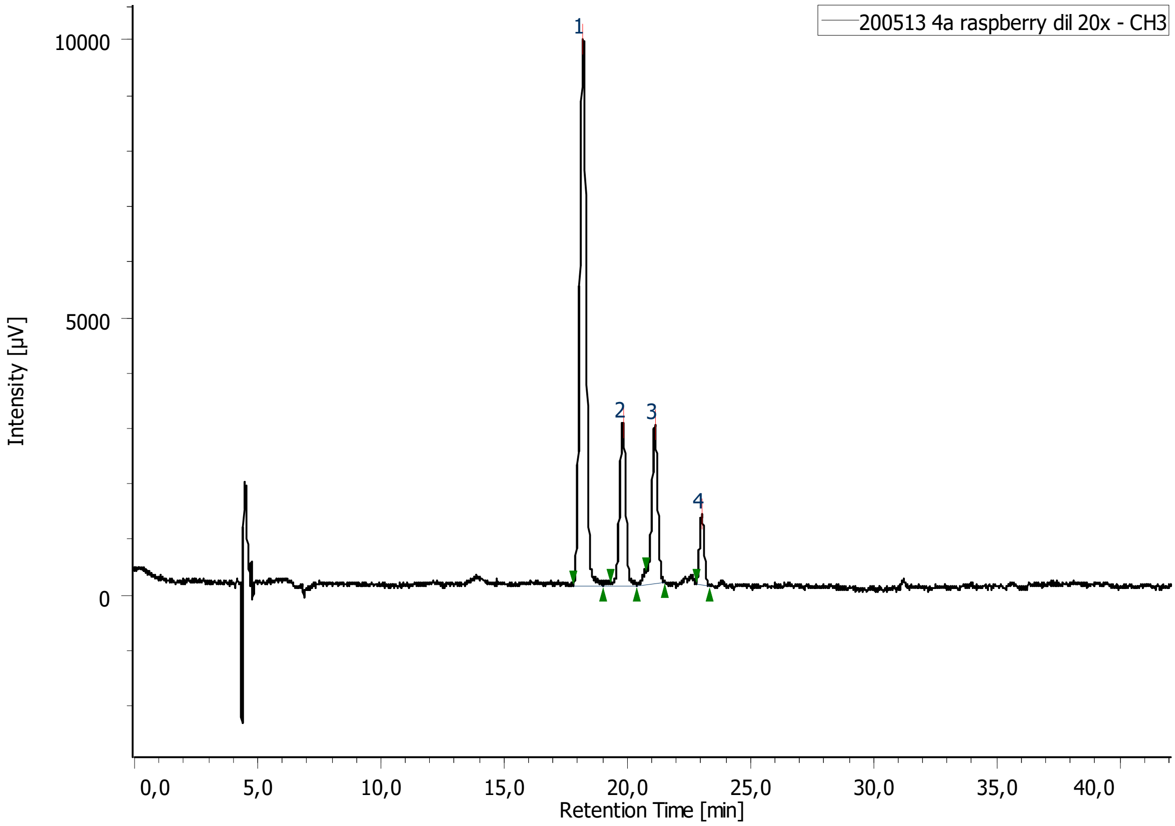

Fig.S1F. Example of a chromatogram for raspberry extract <u>before in vitro digestion</u>. Peaks correspond to the following anthocyanins: (1) cyanidin-3,5-diglucoside, (2) pelargonidin-3-O-rutinoside, (3) delphinidin-3-O-galactoside, (4) delphinidin-3-glucoside, (5) cyanidin-3-O-galactoside.

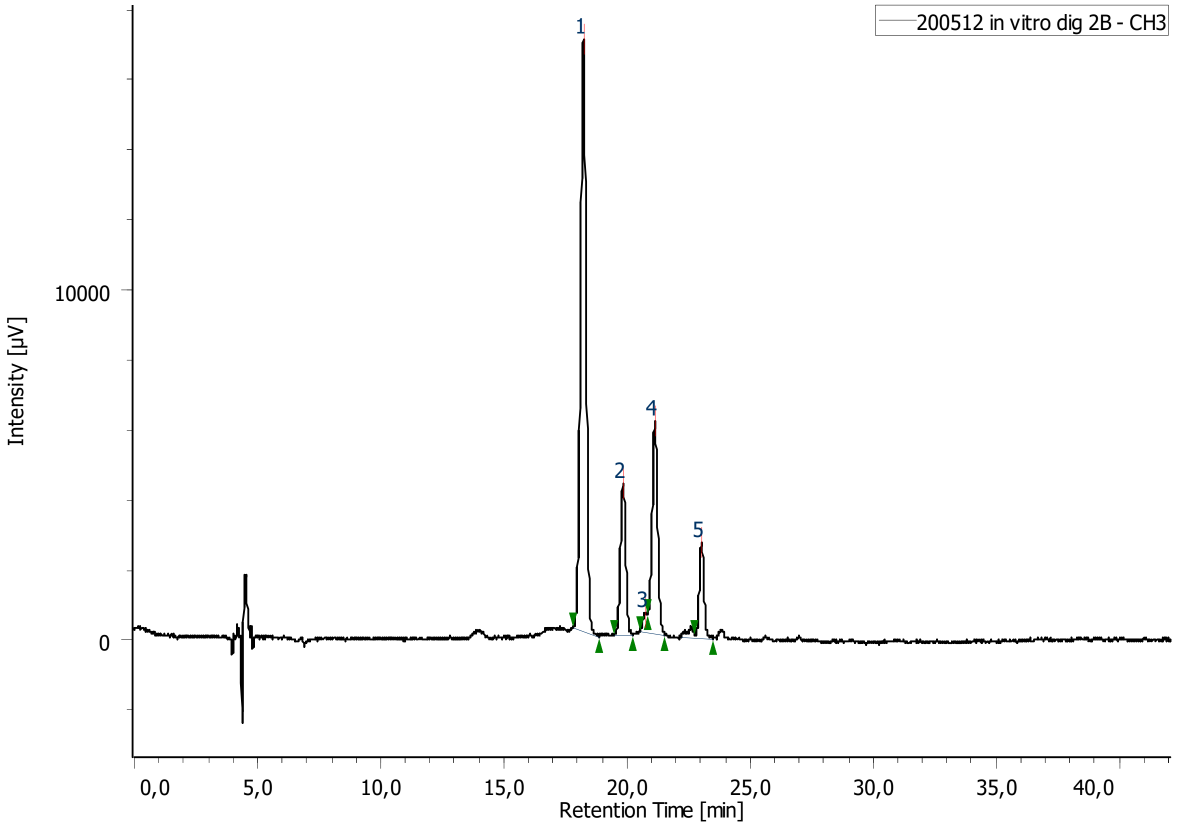

Fig. S1G. Example of a chromatogram for raspberry extract after in vitro digestion. Peaks correspond to the following anthocyanins: (1) cyanidin-3,5-diglucoside, (2) pelargonidin-3-O-rutinoside, (3) delphinidin-3-O-galactoside, (4) delphinidin-3-glucoside, (5) cyanidin-3-O-galactoside.

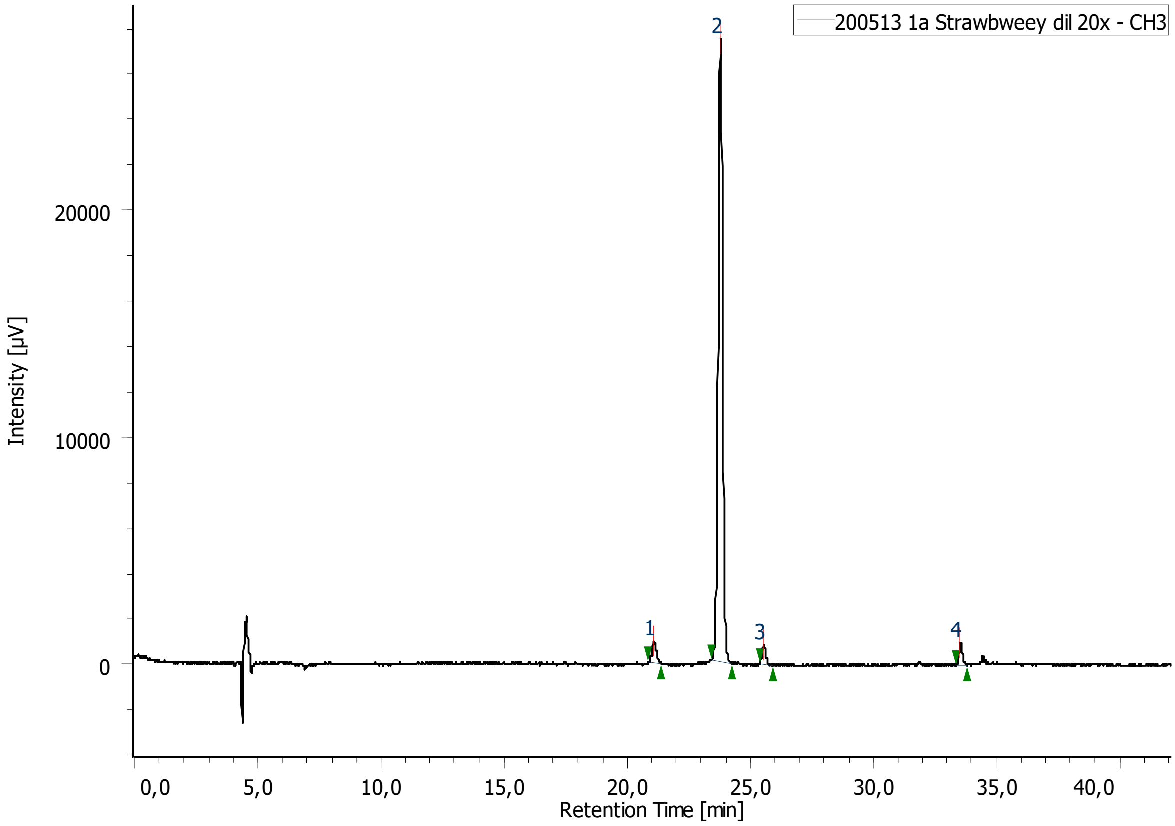

Fig. S1H. Example of a chromatogram for strawberry extract <u>before in vitro digestion</u>. Peaks correspond to the following anthocyanins: (1) cyanidin-3,5-diglucoside, (2) pelargonidin-3-O-rutinoside, (3) delphinidin-3-O-galactoside, (4) delphinidin-3-glucoside.

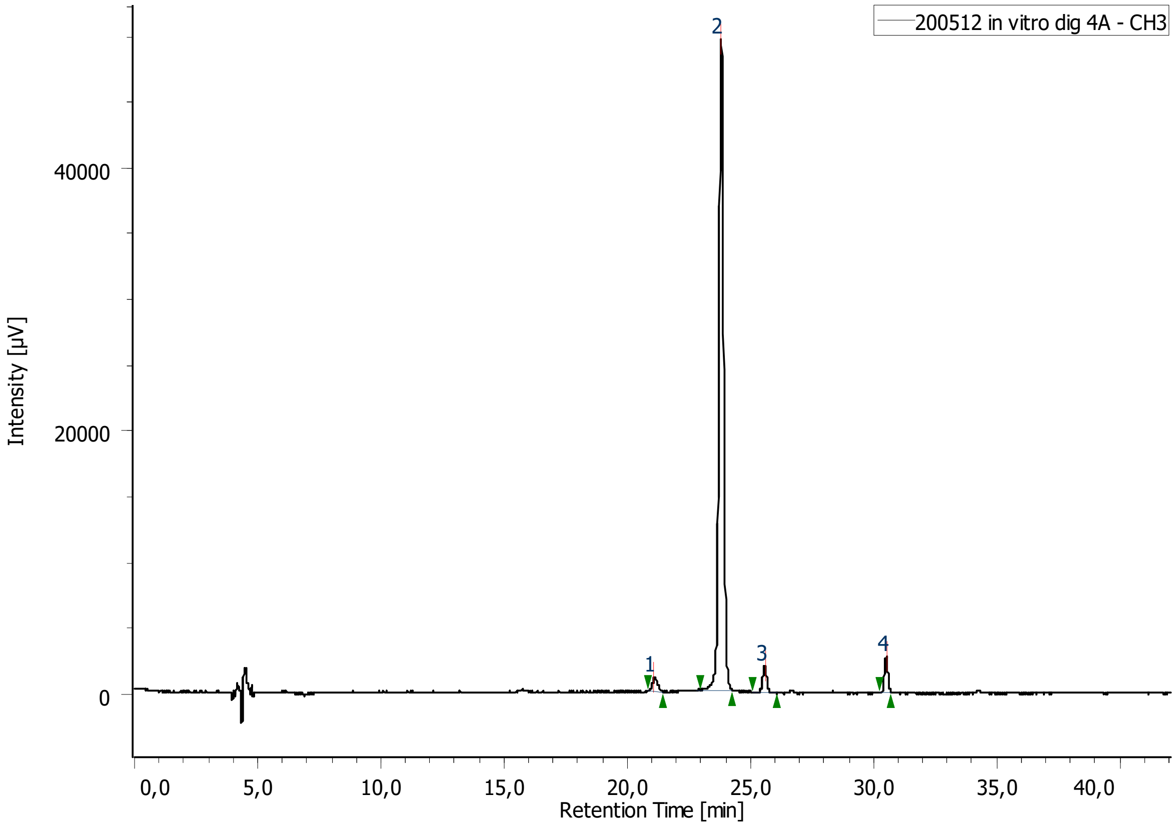

Fig. S1I. Example of a chromatogram for strawberry extract after in vitro digestion. Peaks correspond to the following anthocyanins: (1) cyanidin-3,5-diglucoside, (2) pelargonidin-3-O-rutinoside, (3) delphinidin-3-O-galactoside, (4) delphinidin-3-glucoside.

Table S1. Survival rate averages of *S. cerevisiae* SKQ2n treated with H_2O_2 (0.45 M) cultured in YNB with 2% glucose without berry extracts (Control), with berry extracts before in vitro digestion (before) and after in vitro digestion (After).[a]

Berry	Survival (%)	SE
Bilberry		
Control	17.40 def	5.92
Before	14.05 f	5.67
After	41.27 c	5.83
Blackcurrant		
Control	23.93 de	5.97
Before	15.33 ef	5.86
After	52.16 b	4.47
Raspberry		
Control	19.16 def	5.81
Before	15.84 ef	5.88
After	46.49 bc	6.22
Strawberry		
Control	25.78 d	5.42
Before	22.34 def	5.38
After	79.38 a	2.22

[a]Significant difference between treatments were detected using the Tukey's Studentized Range (HSD) at the 5% level of significance. Averages followed by the same letters are not significantly different (n= 30; p <0.0001). [a]SE= Standard error.
